# The art and science of serious game design: a quasi-experimental study based on self-determination theory in traditional Chinese culture education

**DOI:** 10.3389/fpsyg.2025.1536513

**Published:** 2025-10-27

**Authors:** Jiqiang Yang, Ran Li

**Affiliations:** College of Design and Innovation, Zhejiang Normal University, Jinhua, China

**Keywords:** serious game, traditional Chinese culture, SDT, motivation, game design

## Abstract

**Introduction:**

Drawing upon self-determination theory, this research investigates methods to enhance motivation in educational settings through the use of serious games by addressing the fundamental psychological needs for autonomy, competence, and relatedness.

**Methods:**

Three strategies were proposed to meet these needs: diversifying learning resource presentations, creating appropriate challenges, and integrating relationships into the narrative framework. A serious game incorporating traditional Chinese culture was developed and compared to a short documentary film for learning efficacy. The study involved 60 college students from two natural classes who participated in either a 15-minute gameplay session or a 15-minute video-watching session, followed by a 10-minute discussion. Post-test scores and learning motivation were assessed to evaluate the effectiveness of both methods.

**Results:**

No significant difference in post-test scores was observed between the two groups, indicating that the serious game was as effective as the traditional documentary in enhancing academic performance. However, the serious game significantly outperformed the documentary in enhancing learning motivation, despite being perceived as less authentic. The immersive and interactive elements of the game were found to boost engagement and interest among students.

**Discussion:**

The results underscore the potential of serious games to create engaging and motivating educational environments that cater to diverse student populations while maintaining academic rigor. The findings suggest that serious games can be a valuable tool in education, particularly for enhancing motivation and engagement, even when traditional methods are equally effective in terms of academic performance.

## 1 Introduction

Serious games are games that focus on acquiring knowledge and skill training. They are a type of video and computer game, often used for professional training and education. Serious games may resemble commercial games in appearance and elements, but are not intended for entertainment. By simulating real-life events or processes, users are expected to acquire knowledge, develop abilities, or receive treatment during the gaming experience (Zeng et al., 2020). Following Wouters et al. (2013), we define serious games as digital tools designed for primary educational purposes (e.g., knowledge acquisition, skill development) rather than entertainment. Key traits include: Aligned learning objectives (e.g., historical literacy); Game mechanics mapped to pedagogical goals (e.g., puzzle-solving for critical thinking); Feedback systems for skill mastery (e.g., formative assessments in-game).

After conducting surveys and analyses from the perspective of learning psychology, the bottleneck affecting the effectiveness of traditional cultural course teaching lies in college students’ learning motivation (Li, 2012). Intrinsic conditions (learning needs, the “usefulness” of educational content) and extrinsic conditions interact to influence learning motivation. The teaching content in colleges and universities is too uniform (Zhu, 2021). In the context of informatization, it appears very rigid and stereotyped, making it difficult to attract the attention of young people, leading to insufficient classroom participation. It is evident that the uniformity, rigidity, and dullness of cultural course content lead to low demand for the teaching content by students, lack of interest, poor initiative, and insufficient learning motivation. Therefore, we believe that in order to enhance the appeal of traditional cultural courses to college students, reforms should be made in dimensions such as the presentation of teaching content and enhancing learning motivation. The combination of serious games with traditional cultural education is significantly important for enriching traditional cultural education methods and stimulating students’ learning motivation and interest.

Research on the motivation for serious games in the academic community primarily focuses on improving learning engagement, academic performance/achievement, intrinsic motivation, and extrinsic motivation. Based on literature analysis, research on motivation, engagement, satisfaction, and learning performance related to serious games has been an important research topic in recent decades (Yu et al., 2021). They also noted that there is a positive correlation between motivation and engagement, meaning higher motivation often leads to greater engagement, and vice versa. The purpose of using serious games for learning is to utilize people’s intrinsic motivation to promote learning while playing games (Bourgonjon et al., 2010). Hawlitschek and Joeckel (2017) applied adventure games to the study of “historical events” and examined the impact of learning guidance (as a means of influencing extrinsic motivation) on gamified learning from two dimensions: learning motivation and cognition. The experiment found that the learning guidance in the game had a negative effect on academic performance, yet it did not affect intrinsic motivation. Filsecker and Hickey (2014) conducted an experimental study on the impact of external rewards (such as badges) on elementary students’ learning motivation, engagement, and academic performance in serious games. They discovered that external rewards did not diminish learning motivation and interest, but they also had no effect on improving disciplinary engagement. Additionally, some researches have compared the influence of teacher support and digital support on student engagement in blended learning based on the motivational perspectives of Self-Determination Theory (SDT; Chiu, 2021a; Chiu, 2021b).

Most research on motivation in serious games is based on experimental or quasi-experimental designs (Rahimi et al., 2021; Chowanda and Chowanda, 2016; Minoviæ et al., 2015; Wronowski et al., 2019), case study (Ponticorvo et al., 2019; Callaghan et al., 2018; Kazimoglu, 2020), conducting empirical analyses on learning motivation, academic performance/achievement, etc., and measuring intrinsic motivation from dimensions such as curiosity and interest. The research results can generally be categorized into three types: (1) having a positive effect on intrinsic motivation but no impact on academic performance; (2) having no impact on motivation but positively promoting academic performance/achievement; (3) having a positive effect on both motivation and academic performance/achievement.

Research on the motivation of serious games focuses on the impact of games on learner engagement and learning motivation, as well as the interrelationship between engagement and motivation. Based on this, further studies are conducted to explore the influence of serious games on academic performance/achievement. However, there are several issues that exist:

First, the transformation between intrinsic and extrinsic motivation, such as how external motivation strategies like rewards and badges can promote the maintenance and development of intrinsic motivation, and what implications this has for guiding the design of serious games? Second, what factors influence the engagement with serious games? How do these factors affect “engagement” and in turn impact learning motivation and performance? For instance, factors like visual design and interactive methods.

Third, most empirical research on motivation is conducted in traditional classroom or laboratory environments, with fewer studies exploring motivation from a sociocultural perspective. As a result, external rewards measures, such as coins, health points, and scores, are often critiqued for their potential to undermine intrinsic motivation. This concern arises when such rewards are viewed as controlling or disconnected from the learner’s personal goals. However, from a sociocultural perspective, motivation is better evaluated based on how these rewards support meaningful participation in authentic practices within the target knowledge domain (Hickey and Zuiker, 2005). For instance, game elements like badges or scores can reinforce engagement when they are tied to culturally relevant narratives or tasks that learners find personally meaningful. According to sociocultural theory, well-designed extrinsic motivation does not diminish intrinsic motivation but can instead promote its development when aligned with authentic learning experiences. Therefore, serious games should adopt a multidisciplinary perspective, carefully designing extrinsic rewards to complement intrinsic needs. This approach can sustain learners’ motivation by fostering a sense of autonomy, competence, and relatedness while engaging them in meaningful educational activities.

In the context of traditional culture education, serious games present a novel solution to address longstanding challenges in motivating college students. Traditional methods of teaching cultural content often rely on rigid, uniform presentations that fail to capture students’ interest, resulting in low engagement and limited learning motivation.

Students’ lack of engagement with rigid course content in traditional cultural education underscores the need for innovative approaches. Serious games, with their interactive and immersive qualities, offer a unique opportunity to engage learners by addressing their psychological needs for autonomy, competence, and relatedness. These elements are particularly critical for younger generations, who often find conventional methods unappealing.

Despite the growing interest in serious games for educational purposes, there is limited understanding of their application in traditional cultural education, particularly when designed using SDT principles. SDT emphasizes the fulfillment of psychological needs—autonomy, competence, and relatedness—as key drivers of intrinsic motivation. By embedding these principles into the design of serious games, this study seeks to explore how they can enhance both motivation and learning outcomes in traditional cultural contexts.

This study aimed to (1) evaluate the effectiveness of a serious game, “The Taste of Truth,” in enhancing learning outcomes compared to traditional methods, and (2) examine its impact on learner motivation, engagement, and emotional responses. To achieve these objectives, the study employed a quasi-experimental design involving two groups of college students. The experimental group engaged with a serious game designed on the Roblox platform, while the control group followed a documentary-based instructional method.

The integration of SDT principles into serious game design addresses the motivational challenges of traditional Chinese cultural education, offering a promising pathway to make such content more engaging and impactful. For cultural education (such as history, heritage), serious games can simulate historical decision-making (e.g., “Revolution” game on the American Revolution) or reconstruct cultural contexts (e.g., “Assassin’s Creed: Discovery Tour”). Our game, “The Taste of Truth,” adopted a narrative-driven approach where players interacted with non-player characters (NPCs) based on real historical figures (Petousi et al., 2022; Ye et al., 2021), fostering empathy and contextual understanding.

## 2 Theoretical basis for motivation design in serious games

After comparing 21 of the most popular theories about serious game design, the SDT is by far the most frequently applied theoretical framework (Krath et al., 2021). This is followed by Flow Theory, Constructivist Learning Theory, Experiential Learning Theory, Cognitive Load Theory, and others. SDT originates from the research on intrinsic and extrinsic motivation by Ryan and Deci (1980). Unlike behaviorist theory, which emphasizes external control and influence on motivation, SDT underscores the importance of intrinsic motivation for learning and growth.

The SDT theory highlights the proactive role of individuals in the motivation process, suggesting that human intrinsic motivation can be enhanced by fulfilling three basic psychological needs, thereby promoting the internalization of extrinsic motivation and healthy personal growth. These three fundamental needs are–Autonomy, Competence, and Relatedness (Ryan and Deci, 2000; Ryan and Deci, 2020). “Autonomy” refers to the initiative in action, supported by interest and valuable experiences, not controlled by external conditions such as rewards or punishments. When individuals experience autonomy provided by the external environment, or when they have a high degree of self-determination in activities, they attribute this internally, feeling in charge of their own lives. “Competence” is a feeling of mastery, an awareness of success and growth. A well-structured environment (such as serious games) is more conducive to satisfying the need for competence, offering optimal challenges, positive feedback, opportunities for growth, etc. Similar to Bandura’s concept of self-efficacy, it involves an individual’s belief in their ability to perform at a certain level in their learning behaviors or actions, confident in their competence to undertake the activity (Liu and Zhang, 2010). “Relatedness” refers to a sense of belonging and connection, achieved through respect and care. Any obstacle to these three basic needs is considered detrimental to motivation and health. Therefore, the analysis of educational environments based on SDT primarily focuses on the extent to which these three fundamental human needs—autonomy, competence, and relatedness–are met.

Regarding the transformation between extrinsic motivation and intrinsic motivation, Lee and Hwang pointed out that whether there is a negative effect between intrinsic motivation and extrinsic motivation depends on the type of extrinsic motivation (Lee et al., 2015). Controlled extrinsic motivation undermines intrinsic motivation, but autonomous extrinsic motivation enhances it (i.e., the previously mentioned internalization). Therefore, when designing serious games, one must use external incentives such as scores, badges, coins, and even punishments with caution. How to promote the internalization of extrinsic motivation poses certain challenges in the design of serious games. According to SDT theory (Deci and Ryan, 2008), the transition from extrinsic motivation to intrinsic motivation requires undergoing four states of varying degrees of internalization—from weaker to stronger degrees of internalization: (1) External Regulation, such as external rewards or punishments; (2) Introjection, such as approval from others or oneself; (3) Identification, such as recognizing personal goals, self-worth, etc.; (4) Integration, which is the highest degree of internalization of extrinsic motivation, bearing many similarities with intrinsic motivation, but not intrinsic motivation itself. After transformation through these four states, the final internalization of extrinsic motivation is completed, achieving internal motivation drive. Hence, SDT theory considers the change in motivation to be continuous. The inspiration for the design of serious games is that commonly used game elements like scores, health points (life values), coins, etc., are more rudimentary forms of extrinsic motivation (external regulation) stimuli. We must endow these incentives with certain meanings, satisfying the three basic individual needs, to avoid extrinsic motivation remaining in a lower (external regulation) state.

## 3 Motivation design strategies for serious games

In video gaming industry, traditional cultural elements have been fully utilized. This integration not only adds cultural connotations and artistic value to the games themselves but also promotes the dissemination and innovative transformation of traditional culture. Especially in the context of the digital era, this trend is becoming increasingly evident. As a convergence point of modern technology and culture, video games provide a new platform for the inheritance of traditional culture through their interactivity and immersion. For example, the game “Genshin Impact” combines traditional Chinese culture with gameplay through virtual regions like “Liyue” and festivals like the “Lantern Rite,” with extensive use of traditional Chinese architectural styles in the game scenes. “Honor of Kings” incorporates a large number of intangible traditional Chinese cultural elements into its skin designs, such as Dunhuang Feitian, Yue Opera, Su Embroidery, etc. which not only enriches the cultural connotations of game characters but also allows young players to encounter and understand these precious cultural heritages while playing the game.

Unlike the utilitarian and commercial aspects of typical video games, serious games are a type of computer software designed with specific educational goals. These games have the ability to simulate real-life scenarios, stimulate learners’ intrinsic motivation, and facilitate learning outcomes through an engaging and enjoyable experience (Dörner et al., 2016). Serious games are developed for the sake of specific educational purposes (Djaouti et al., 2011), incorporating more traditional cultural elements into scene design can enhance their educational functions and values, thereby improving educational effectiveness. Many digital game platforms (software), such as Roblox, offer a rich terrain system, realistic modeling scenes, and interactive design methods, providing necessary support for game design. Currently, the most pressing research question for serious games should not be whether games can facilitate learning, but how to fully engage students in learning through design (Nousiainen, 2009; Lacasa et al., 2009). According to SDT, when three basic needs (autonomy, competence, relatedness) are satisfied, external motivation becomes “internalized,” and intrinsic motivation is enhanced. Therefore, for traditional cultural courses, exploratory game activities can be designed where individuals experience “autonomy” through free exploration in a virtual gaming environment and “competence” by overcoming challenges in the game. They accomplish the learning of more tedious theoretical knowledge through genuine or virtual interactions with classmates, partners, or NPCs. So, this study designed a traditional cultural serious game from the following three aspects to promote users’ motivation.

### 3.1 Integrating “relatedness” into the history context–meeting the need for “relatedness”

Although the need for relatedness is more distal than the needs for autonomy and competence in fostering intrinsic motivation, it is more central in promoting the internalization of extrinsic motivation (Zhang et al., 2010). By designing the storylines of the game, “relatedness” can be blended with historical and cultural backgrounds, thereby enhancing the realism and sense of reality of the game. Therefore, we conducted field research on the hometown of the protagonist of our game - Chen Wangdao, and made detailed observations of the local customs, geography, human environment, and traditional architecture.

Serious games with storylines can enhance students’ immersion and sense of identification (He and Dong, 2021). Social disciplines emphasize emotional exchange and communication, which can be achieved through role-playing games by simulating real-life scenarios via scene design. We designed storylines such as how Chen Wangdao prepared for studying abroad (e.g., finding a land deed to sell the land), receiving the translation task from Mr. Shao Lizi after returning from studying abroad, and searching for the key to the woodshed in the sugarcane forest. Overall, this game allows students to experience the scenery, folk customs, and traditional residential culture of Chen Wangdao’s hometown through digital storytelling and relationship building, from a first-person perspective (RPG game mode).

According to SDT theory, a player’s perception of their relationship with virtual characters significantly impacts the success or failure of game design (Oliver et al., 2016). To enhance the realism of traditional cultural games, we primarily designed three sets of relationships: father-son (such as Chen Wangdao and his father), friendship (such as Chen Wangdao and Shao Lizi), and stranger relations. These three relationships correspond to functions in the game such as navigation, mission guidance, and implicit knowledge transfer, respectively. These NPCs can also provide players with informative rewards, namely positive feedback, thereby establishing a sense of mutual respect and reliance with others (based on virtual characters designed from real individuals). Through the Roblox platform, this game can be designed in a multi-player mode, thus constructing both real relationships with fellow players and virtual relationships with NPCs in the game.

### 3.2 Diversification of learning resource presentation - achieving the need for “autonomy”

Li et al. (2020) examined how autonomous options in game design affect the intrinsic motivation in gamified learning. Providing autonomous options in games is a specific application of SDT theory. In our game, we primarily present learning resources through NPC dialogues, question integration, and visualizing implicit knowledge.

First, it is achieved through player dialogue with NPC characters. Game NPCs or “helpers” can be used to implement scaffolding and coaching strategies (Shang et al., 2012). For example, in the dialogue with the NPC character Shao Lizi, we incorporate historical knowledge, background, and processes, allowing learners to understand corresponding historical and cultural knowledge through interaction with NPCs.

Second, we employ a subtle question integration strategy. In traditional serious games, learners answer questions to receive corresponding “rewards,” and upon collecting enough rewards, they advance to the next level. We believe this method can disrupt the sense of immersion and flow experience in gaming, to some extent depriving learners of “autonomy.” Therefore, we adopted a strategy of subtly integrating questions into gameplay. For example, we designed the sugarcane field as a maze puzzle, with key positions within the maze incorporating relevant questions (with built-in feedback functions). This design ensures that learners do not feel like they are answering questions but rather navigating the maze. All questions are not presented in a simple question-stem-and-answer format but are visually designed to blend with the environment.

**FIGURE 1:**
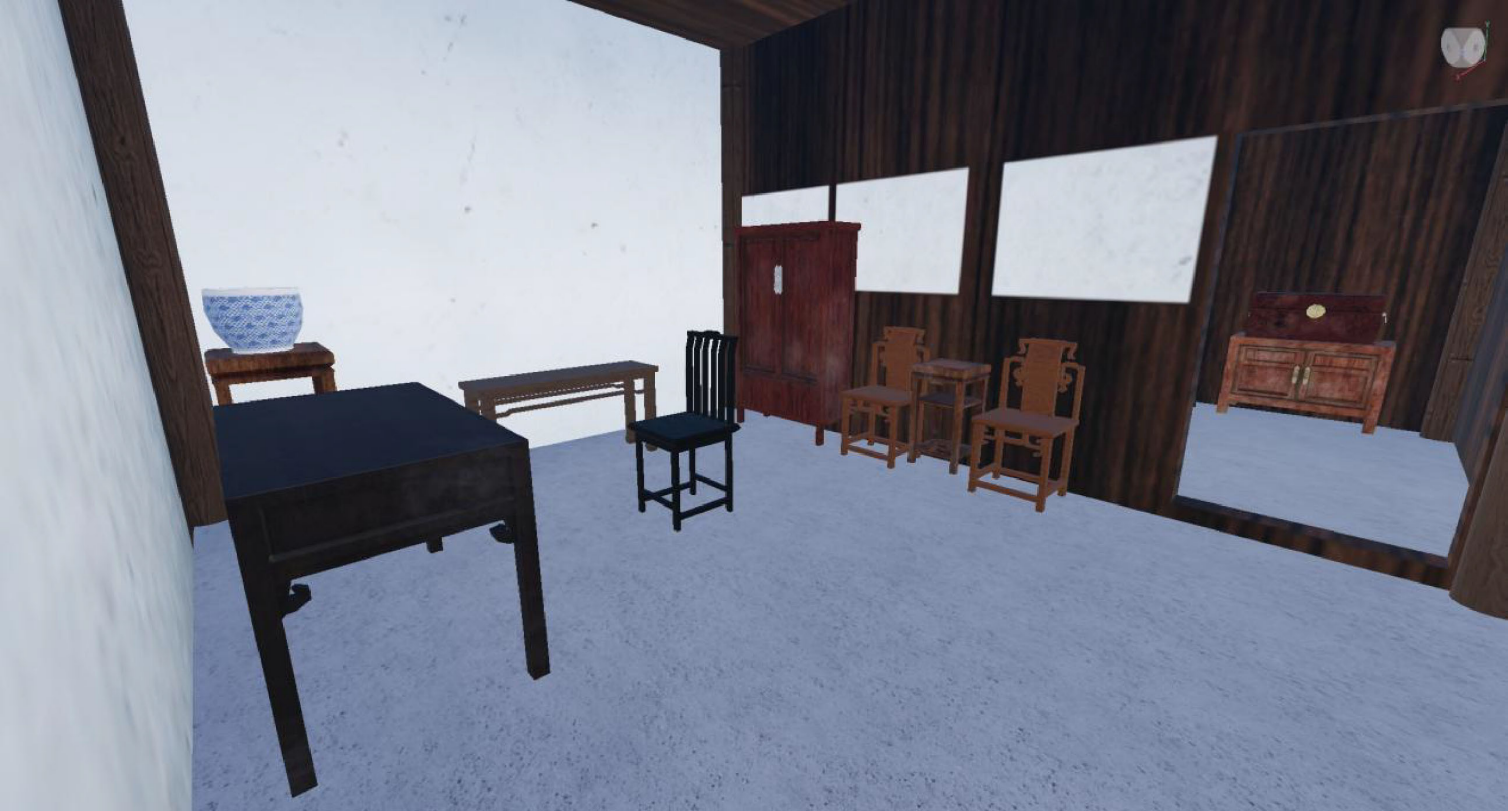
Interior design screenshot of “The Taste of Truth.”

**FIGURE 2:**
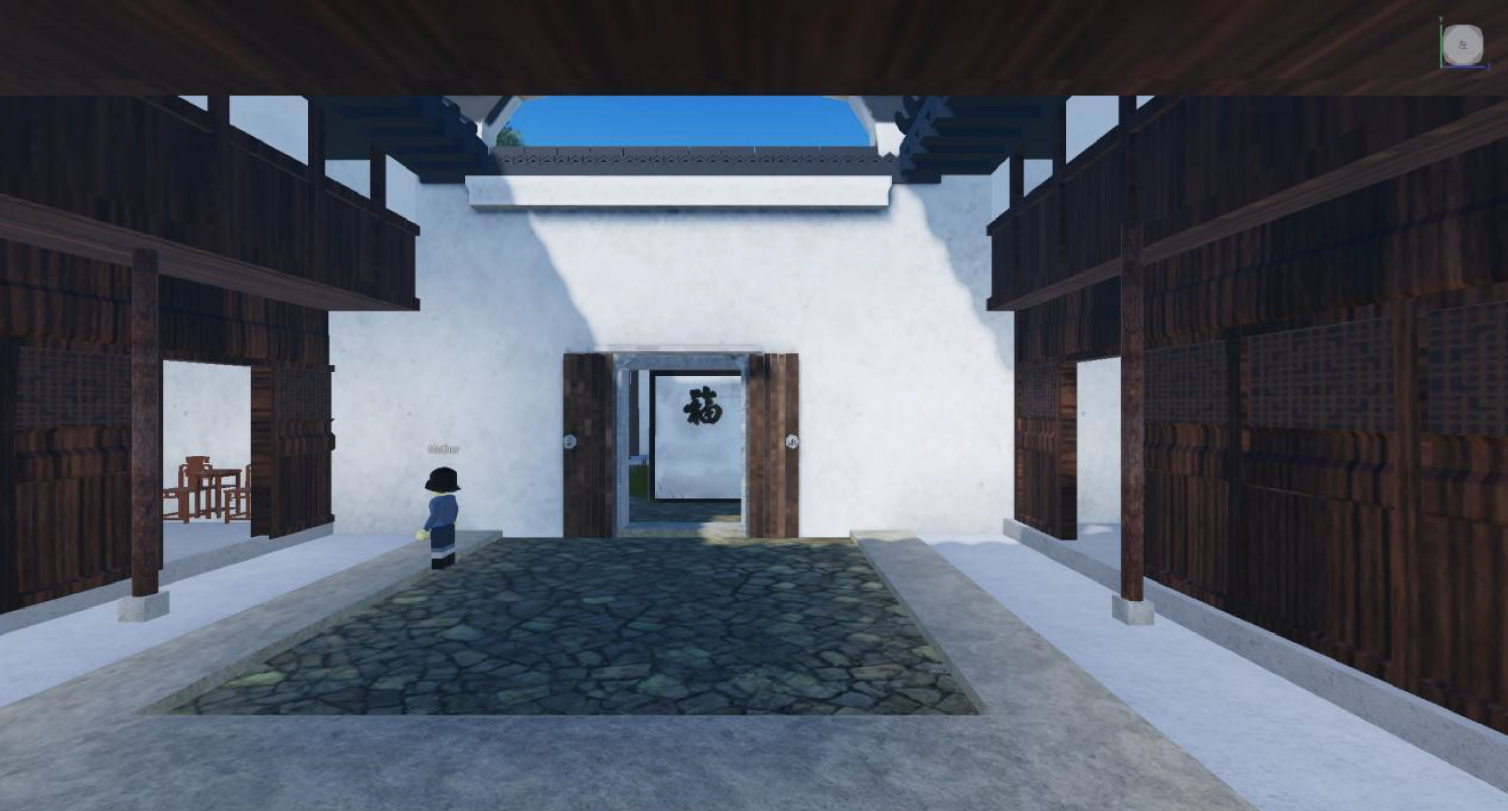
Interior screenshot of the game architecture.

Finally, we integrate implicit knowledge into the game scenarios for learners to experience autonomously. Factors such as resource availability and interactive design in the learning environment can influence learner engagement (cognitive, behavioral, and emotional engagement) (Kamkuimo et al., 2020). In traditional culture-themed games, the beauty of one’s hometown is an implicit form of knowledge; an aesthetically pleasing environment can cultivate individuals’ character. Kuksa advised to reshape the learning spaces by blending the virtual and physical realms, providing students with authentic experiential environments (Kuksa and Childs, 2014). We extracted elements from Chen Wangdao’s hometown, such as sugarcanes, architecture, streams, rice fields, and village layouts, and artistically presented these elements in the game visuals (Figures 1, 2). By organically integrating implicit knowledge about local specialties, products, landscapes, geography, and cultural information into the visual design of the game, we not only enhance the sense of immersion in traditional culture games but also reshape the learning space. This provides learners with a gamified learning space that blends the virtual and real, allowing for autonomous experiences in an interactive virtual environment and achieving the effect of cultural education subtly.

Additionally, the game also included designs for free exploration and decision-making, as well as non-linear narrative and tasks. The tasks and interactions within the game were designed to allow flexibility in task completion order. Players could decide which challenges to tackle first, fostering a sense of control and self-direction. This gave users a sense of ownership over their learning experience.

### 3.3 Creating appropriate “challenges”-meeting the need for “competence”

The most commonly used game elements in serious games are, in order: feedback, challenge, score, collaboration, rule, role-playing, etc., and pointed out that “penalty” must be specific in an educational gaming environment and is not a common element (Noroozia et al., 2020). In the design of traditional Chinese culture games, we mainly set two types of challenges: complex and simple. Simple challenges include finding the key to the woodshed, finding the land deed, etc., while more complex challenges include navigating through a maze, crossing a shaking broken bridge, etc. After completing these challenges, players will receive corresponding score rewards. Conway found that rewards set to motivate players’ extrinsic motivation (such as scores, gold coins, etc.) are important factors in promoting players to level up or complete levels in games. However, if this process is given some meaning, it will be more conducive to stimulating players’ intrinsic motivation and promoting the internalization of extrinsic motivation (Conway and Elphinstone, 2017). To achieve this transformation, we designed a plot where players search for land deeds, sell land to raise tuition fees, and receive score rewards. Through such design, the game activities are endowed with “real” significance - studying abroad and returning to serve the country after graduation. In students’ real social lives, they have the desire to study abroad and to earn material rewards, and the appropriate “challenging” tasks in the game, such as finding land deeds, selling land, obtaining gold coins, and studying abroad, also effectively meet the contemporary college students’ real needs. That is to say, when the design successfully connects with the player’s Dasein, the sense of relatedness to the entire gameworld is augmented (Conway and Elphinstone, 2017).

Based on the above design strategies, we developed a serious game: “The Taste of Truth” on the Roblox platform.

Above all, by addressing autonomy, competence, and relatedness through these tools, the study demonstrated how serious games could foster intrinsic motivation and engagement. These strategies align with SDT principles and highlight the potential of gamified learning environments to create meaningful educational experiences.

## 4 Materials and methods

This study was approved by the Ethics Committee of Zhejiang Normal University (Approval No: ZSRT2025123), Written informed consent was obtained from all participants.

### 4.1 Subjects and grouping

Two natural classes of college students majoring in Digital Media Art were selected for this quasi-experimental study. The classes were chosen based on similar demographics (gender balance, major) and prior learning outcomes to minimize confounding variables.

Each class was randomly assigned as either the experimental group (game-based class) or the control group (traditional class). Both groups consisted of 30 students, balanced in gender distribution (Experimental: 15 male, 15 female; Control: 14 male, 16 female).

### 4.2 Materials

Game-based class

The serious game “The Taste of Truth” incorporated traditional Chinese cultural elements designed according to SDT principles:

(1) Autonomy: Players navigated a sugarcane maze, interacted with NPCs, and uncovered historical knowledge through non-linear storytelling.
(2) Competence: Tasks included retrieving a land deed, solving puzzles, and earning rewards, designed to balance difficulty and achievement.
(3) Relatedness: Emotional and narrative elements were introduced through interactions with NPCs representing familial and social relationships.

Traditional class:

The control group watched a documentary for 15 min, “Chen Wangdao’s Hometown,” which covered the same content as the game.

### 4.3 Procedures

The experiment involved the following steps:

(1) Pre-test: administered 2 weeks before the intervention to assess students’ baseline knowledge of the subject.
(2) Intervention:
Game-based class: students played the game for 15 min, followed by a 10-min group discussion led by the instructor.
Traditional class: the students watched the documentary for 15 min. Next, the students and their teacher same with the experimental group engaged in a 10-min discussion about the cultural information presented in the video.
(3) Post-test: administered 1 week after the intervention to evaluate knowledge retention and application.
(4) Questionnaire: assessed dimensions like motivation, immersion, and interactivity etc.
(5) Control variables: demographic data (gaming experience, cultural interest, digital literacy) were collected to analyze external influences on learning outcomes and motivation.

### 4.4 Instruments

(1) Pre-test and Post-test:
  10 multiple-choice questions covering factual recall, conceptual understanding, and cultural application, each scored out of 100. The pre- and post-tests were reviewed by two experts in educational psychology for content validity.
(2) Questionnaire:
  A 12-item survey measuring four dimensions: game navigation, immersion, interactivity, and motivation (Lafrenière et al., 2012; Huang et al., 2010; Eppmann et al., 2018; Johnson and Norris Martin, 2014; Norris et al., 2014). Items were scored using a 5-point Likert scale. Cronbach’s Alpha for reliability was 0.812. See Appendix A for full items, and Appendix B for the documentary group.
(3) External variables:
  Gaming experience, cultural interest, and digital literacy were measured using self-reported scales and short proficiency tests. Results were categorized into low, medium, and high levels. Preliminary analysis showed that higher gaming experience correlated with faster adaptation to the serious game mechanics (mean completion time of maze challenge: low experience = 12 min; high experience = 7 min). Similarly, those with medium to high interest in traditional culture reported higher motivation scores in both groups.

### 4.5 Additional metrics

(1) Engagement

Time-on-task: The duration participants spent interacting with the serious game or watching the documentary.
Navigation Patterns: Interaction logs from the game platform were analyzed to track movement through challenges, task completion rates, and pauses.

(2) Cognitive load

1. Participants completed the NASA-TLX (Task Load Index) questionnaire after the intervention to measure their perceived cognitive load during the learning activity. Lower cognitive load scores indicated a smoother learning experience.

(3) Emotional responses

1. A 6-item self-report scale was used to capture emotional engagement, with questions like “Did you enjoy the learning experience?” and “How curious did you feel about exploring the content?” Scores ranged from 1 (Not at all) to 5 (Very much).

### 4.6 Hypotheses

(1) There is no difference between game-based and traditional classes in improving learning outcomes in traditional culture learning.
(2) There is no difference between game-based and traditional classes in enhancing learners’ motivation in traditional culture learning.

## 5 Results and discussion

### 5.1 Result

#### 5.1.1 Post-test analysis

Table 1 presents the results of the independent samples *t*-test on post-test scores between the experimental group (serious game) and the control group (documentary). The experiment, through a 15-min intervention (game/video) followed by a 10-min discussion, aimed to verify the effectiveness of the two methods in enhancing participants’ knowledge of traditional Chinese culture. The results showed no significant difference in post-test scores between the two groups (*p* > 0.05), indicating that serious games are academically equivalent to traditional approaches in performance.

**TABLE 1:** Post-test score independent samples *t*-test.

Groups	Mean	Std. Deviation	*N*	95% CI	*F*	*P*
Game-based	80.00	10.17	30	[76.30, 83.70]	0.064	0.801
Traditional	79.67	10.33	30	[75.90, 83.40]		

#### 5.1.2 Engagement and emotional responses

Table 2 compares the two groups’ performance in engagement (task duration) and emotional responses (enjoyment/curiosity). The game group’s task duration was nearly 200% that of the traditional group, with significantly higher self-reported enjoyment (mean 4.2/5) and curiosity (mean 3.6/5), proving that serious games substantially enhance learning engagement and emotional experiences.

**TABLE 2:** Engagement and emotional response metrics

Dimension	Metric	Game-base class	Traditional class
Engagement	Average time-on-task (min)	15	8
Cognitive load	NASA-TLX Score (0–100)	59	50
Emotional responses	Self-reported enjoyment (1–5)	4.2	3.6

#### 5.1.3 Motivation analysis

Table 3 contrasts the groups’ scores on the motivation scale (participation duration/cognitive load/emotional enjoyment). The game group scored significantly higher in total motivation than the traditional group (*p* < 0.05).

**TABLE 3:** Motivation scores.

Groups	Mean	Std. Deviation	*N*	95% CI	*P*
Game-based	10.57	2.20	30	[9.76, 11.38]	0.000***
Traditional	8.13	1.45	30	[7.59, 8.67]	

****Indicates a statistically significant result at a very high level of confidence.

### 5.2 Discussion

#### 5.2.1 Hypothesis 1: learning outcomes

The lack of significant difference in post-test scores suggests that serious games are as effective as traditional methods in facilitating knowledge retention and understanding. This aligns with prior research emphasizing the educational potential of serious games without compromising academic Rigor. Several factors may explain why the game group did not significantly surpass the documentary group: (1) the short intervention duration limited opportunities for knowledge consolidation, (2) the assessment focused on factual recall rather than deeper conceptual understanding, and (3) the traditional group’s passive instruction may have been more effective for short-term memorization. These findings align with SDT’s emphasis on motivation as a precursor to, but not a guarantee of, learning success (Ryan and Deci, 2000). Future designs could integrate SDT principles with explicit instructional strategies (e.g., in-game formative assessments) to bridge the motivation-learning gap.

#### 5.2.2 Hypothesis 2: learner motivation

Contrary to the hypothesis, serious games significantly outperformed traditional methods in motivating learners. Immersive elements, interactive challenges, and emotional connections contributed to increased engagement, supporting SDT’s premise that fulfilling autonomy, competence, and relatedness boosts intrinsic motivation.

On the one hand, The immersive and interactive elements of serious games likely contribute to increased engagement and interest, which are crucial components of motivation. These findings are consistent with SDT, which posits that autonomy, competence, and relatedness are key drivers of motivation and can be effectively supported through gamified learning environments (Ryan and Deci, 2000). On the other hand, Unlike Moradi and Noor’s (2022) PBL-SG framework, which focused on abstract computer graphics concepts, our game integrated SDT principles with culturally specific narratives. This suggests that SDT-based design may need cultural adaptation to maximize motivation in heritage education.

While the superior motivation scores in the game group support SDT’s relevance to serious game design, this study acknowledges SDT’s limitations in gamified contexts. For example, SDT’s focus on intrinsic motivation may overlook the complementary role of extrinsic rewards (e.g., badges) in short-term engagement (Hamari and Koivisto, 2015). Additionally, our 15-min intervention may not capture long-term motivation decay, which is different from studies such as Fathali and Okada (2017) who examined SDT effects after prolonged exposure. Future research should integrate hybrid models (e.g., SDT + Flow Theory) to address these gaps.

## 6 Conclusion

Participants in the serious game outperformed traditional teaching methods in learning motivation (especially autonomy and interactivity) and engagement (time-on-task and emotional investment), while both approaches showed comparable effectiveness in knowledge retention and understanding. Our study bridges SDT and game design research by providing empirical evidence that culturally grounded narratives and interactive mechanics can operationalize psychological needs. This aligns with Ryan and Deci’s (2020) call for “contextually sensitive” interventions, suggesting that SDT-based serious games may serve as a scalable model for cultural education.

The integration of SDT principles into serious game design not only explains the observed motivation gains but also provides a theoretical lens for future cultural education interventions. By systematically addressing autonomy through choice, competence through challenge, and relatedness through narrative, our study demonstrates how game mechanics can transcend mere entertainment to become pedagogical tools. These findings align with recent advances in SDT-based gamification (Rodriguez-Calzada et al., 2024) and problem-based learning (Moradi and Noor, 2022), suggesting that a needs-supportive design approach may be universally applicable across disciplines.

### Limitations and future research

The study is limited by its sample size and short intervention duration, which may restrict the generalizability of the findings. Future research could expand sample diversity, extend intervention periods, and explore how serious games integrate with other teaching modalities.

## Data Availability

The raw data supporting the conclusions of this article will be made available by the authors, without undue reservation.

## Appendix

**Appendix A:** Questionnaire for game group.

Dimension	Item
Navigation	1. The game interface clearly indicated my current objectives
	2. I could easily find information to progress.
	3. The menu and controls were intuitive.
Immersion	4. I felt absorbed in the cultural storyline.
	5. Time passed quickly while playing.
	6. The environment connected me to the culture.
Interactivity	7. My actions directly influenced outcomes.
	8. The game provided immediate feedback.
	9. I had meaningful choices to customize my path.
aMotivation	10. The game sparked my curiosity about the culture.
	11. I felt accomplished completing challenges.
	12. I would recommend this game for cultural learning.

Scale: 1 = strongly disagree, 2 = disagree, 3 = neutral, 4 = agree, 5 = strongly agree.

**Appendix B:** Questionnaire for documentary group.

Dimension	Item
Cognitive immersion	1. I was completely absorbed in the cultural content due to the documentary’s narrative.
	2. I was completely absorbed in the cultural content due to the documentary’s narrative.
	3. The documentary’s scenes made me feel connected to traditional culture.
Cognitive engagement	4. The documentary prompted me to actively reflect on the cultural knowledge.
	5. The information in the documentary was presented clearly.
	6. I would explore more about the cultural themes after watching.
Motivation	7. The documentary sparked my curiosity to learn more about traditional culture.
	8. Understanding the cultural content gave me a sense of accomplishment.
	9. I would recommend this documentary for cultural learning.

Scale: 1 = strongly disagree, 2 = disagree, 3 = neutral, 4 = agree, 5 = strongly agree.
